# Special Issue: The Science and Technology of 3D Printing

**DOI:** 10.3390/ma14216261

**Published:** 2021-10-21

**Authors:** Tuhin Mukherjee

**Affiliations:** Department of Materials Science and Engineering, The Pennsylvania State University, University Park, PA 16802, USA; tuhin@psu.edu

## 1. Introduction

Additive manufacturing, commonly known as three-dimensional printing (3D printing), is becoming an increasingly popular method for making components that are difficult to fabricate using traditional manufacturing processes. It enables a one-step fabrication of complex parts directly from a 3D design. 3D printed parts are now regularly used in medical, aerospace, automotive, energy, marine, and consumer product industries [1]. Examples of printed parts include patient-specific, customized medical implants; aero-engine components; parts with complex, intricate features and internal channels; lattice structures; and materials with site-specific chemical compositions, microstructures, and properties [2]. These parts are printed using metallic alloys, polymers, ceramics, and composites. However, the printing of metals and metallic alloys is the fastest developing field because of its applications, demand, and ability to print unique, functional parts. Depending on the material, geometry, and complexity of the part, several 3D printing processes can be employed [2]. For example, for printing metallic parts, powder bed fusion and directed energy deposition processes are commonly used. Thin layers of the powder of wire feedstocks are melted using a high-energy laser, electron beam, or electric arc, which form the part after solidification. Similarly, several processes are used in the industry to print parts with polymers, ceramic, and composites.

Several scientific and technological aspects of 3D printing processes are poorly understood [1]. For example, metal printing involves rapid melting, heat transfer, the convective flow of liquid metal, solidification, and cooling, all of which affect the part’s geometry, microstructure, and properties [2]. Depending on the printing process, materials, and processing conditions, the cooling rates, temperature gradient, and solidification growth rates may vary significantly, which can produce a wide variety of grain structures, morphologies, and textures. Printed parts often suffer from defects such as porosity and cracking that degrade the mechanical properties, quality, and serviceability of the components. In addition, process planning and control to increase productivity without affecting the part quality is a challenging task. All of the scientific and technological issues of 3D printing, as discussed, affect the cost and market penetration of printed parts.

Research and development projects are being performed worldwide to provide a better understanding of the science and technology of 3D printing to make high-quality parts in a cost-effective and time-efficient manner. This Special Issue includes contemporary, unique, and impactful research on 3D printing from leading organizations worldwide.

## 2. Contributions to This Special Issue

This Special Issue contains eleven articles, including three reviews [3,4,5], one perspective article [6], and seven research articles [7,8,9,10,11,12,13] from leading institutes in the United States, China, Australia, Germany, Sweden, the Netherlands, Slovakia, the Czech Republic, Egypt, and United Arab Emirates. These articles cover the 3D printing of diverse materials such as metallic materials [5,7,8,9,10,11,12,13], composites [4,6], and soft materials [3].

The articles in this Special Issue cover a wide variety of experimental [8,9,10,11,13], theoretical [12], and data-science [7]-based research on the science and technology of 3D printing. For example, experimental investigations were performed to identify the most important factors that affect the microstructure and properties of Ti-6Al-4V parts printed using powder bed fusion [9,11]. In particular, the effects of powder morphology, preheating temperature, and post-process heat treatment on the size of α-grains, the hardness, and the tensile properties were studied. In addition, formation mechanisms of defects such as pores and cracks, and their harmful effects on the mechanical properties of printed parts were investigated [8,10,12,13]. A high-speed synchrotron X-ray imaging technique [10] was used to reveal the mechanisms of the evolution of pores during powder bed fusion. A new computer-aided quality (CAQ) technology [13] was proposed and used to determine the amount of porosities in the parts printed using powder bed fusion. The mechanical properties of stainless steel and titanium alloy parts were shown to be affected by the presence of pores [8]. A theoretical model was proposed and used to identify the conditions of crack growth and to discuss the detrimental effects of cracking on the mechanical properties of printed parts [12].

The three reviews and the perspective article indicate the progress made, the existing challenges, and the research needs in the contemporary fields of study. Recent trends and innovations in the printing of soft materials for wearable devices, soft robotics, and tissue engineering were reviewed by Regis et al. [3]. Pervaiz et al. [4] reviewed the 3D printing of fiber-reinforced plastic composites using fused deposition modeling. In fiber-reinforced plastic composites, fibers are mixed in a polymeric matrix. These composite materials are printed to make parts for the defense, automotive, aerospace, and sports equipment industries. The review also emphasized several challenges in the printing of these composites that need to be solved to increase market penetration. The printing of metallic materials allows for the fabrication of parts with unique chemical compositions using elemental powder blends. Chen et al. [5] critically reviewed the progress made in this area and emphasized several critical technical challenges that require further research. Finally, a perspective on the printing of tools for carbon fiber-reinforced composite applications using the binder jet process was provided [6].

## 3. Summary and Outlook

The eleven articles published in this Special Issue focus on the recent advancements in the 3D printing of metallic materials, composites, and soft materials. The achievements reported in this Special Issue are unique, high-quality, and impactful. Due to the wide variety of published work, including experimental, theoretical, and data science-based research, this Special Issue would be of significant and immediate interest to a diverse materials community.

Apart from the exciting advancements, the articles of this Special Issue also identified several scientific, technological, and economic challenges that need immediate attention. The articles pointed towards future research and development that are necessary to print high-quality parts in a cost-effective manner. Clearly, the work in these important areas of 3D printing is just beginning and several decades of research are needed to make 3D printing commercially viable for small and medium-sized companies worldwide.
